# Partially covered versus uncovered pyloro‐duodenal stents for unresectable malignant gastric outlet obstruction: Randomized controlled study

**DOI:** 10.1111/den.14650

**Published:** 2023-08-24

**Authors:** Anthony Yuen Bun Teoh, Sundeep Lakhtakia, Damien Meng Yew Tan, Stefano Francesco Crinò, Vinay Dhir, Rastislav Kunda, Tiing Leong Ang, Khek Yu Ho, Maridi Aerts, Sana Fathima Memon, Shannon Melissa Chan, Philip Wai Yan Chiu, Maria Cristina Conti Bellocchi, Nouredin Messaoudi, Stephen Ka Kei Ng, Hon Chi Yip, Armando Gabbrielli, Christopher Jen Lock Khor, Mohan Ramchandani, Enders Kwok Wai Ng

**Affiliations:** ^1^ Department of Surgery, Prince of Wales Hospital The Chinese University of Hong Kong Hong Kong SAR China; ^2^ Department of Gastroenterology Asian Institute of Gastroenterology Hyderabad India; ^3^ Institute of Digestive and Liver Care SL Raheja Hospital Mumbai India; ^4^ Department of Gastroenterology and Hepatology Singapore General Hospital and Duke‐NUS Medical School Singapore City Singapore; ^5^ Department of Gastroenterology and Hepatology Changi General Hospital Singapore City Singapore; ^6^ Department of Medicine National University of Singapore Singapore City Singapore; ^7^ Gastroenterology and Digestive Endoscopy Unit, Pancreas Institute University Hospital of Verona Verona Italy; ^8^ Department of Surgical Gastroenterology Aarhus University Hospital Aarhus Denmark; ^9^ Department of Surgery, Hepatopancreatobiliary Center Universitair Ziekenhuis Brussel, Vrije Universiteit Brussel Brussels Belgium; ^10^ Department of Gastroenterology‐Hepatology, Hepatopancreatobiliary Center Universitair Ziekenhuis Brussel, Vrije Universiteit Brussel Brussels Belgium

**Keywords:** malignant gastric outlet obstruction, partially covered duodenal stent, uncovered duodenal stent

## Abstract

**Objectives:**

The aim of the current study was to compare the efficacy of partially covered duodenal stent (PCDS) vs. uncovered duodenal stent (UCDS) in patients suffering from unresectable primary malignant gastric outlet obstruction (GOO).

**Methods:**

This was a prospective international randomized controlled study conducted in 10 high‐volume institutions. Consecutive patients suffering from malignant GOO were recruited. The primary outcome measurement was the reintervention rate. Secondary outcomes included technical and clinical success, 30‐day adverse events, 30‐day mortality, causes of stent dysfunction, and the duration of stent patency.

**Results:**

Between March 2017 and October 2020, 115 patients (59 PCDS, 56 UCDS) were recruited. The 1‐year reintervention was not significantly different (PCDS vs. UDCS = 12/59, 20.3% vs. 14/56, 25%, *P* = 0.84). There was a trend to fewer patients with tumor ingrowth in the PCDS group (6/59 [10.2%]) vs. 13/56 [23.2%], *P* = 0.07). There were no significant differences in the technical success (100% vs. 100%, *P* = 1), clinical success (91.5% vs. 98.2%, *P* = 0.21), procedural time (21.5 [interquartile range [IQR] 17–30] vs. 20.0 [IQR 15–34.75], *P* = 0.62), hospital stay (4 [IQR 3–12] vs. 5 [IQR 3–8] days, *P* = 0.81), 30‐day adverse events (18.6% vs. 14.3%, *P* = 0.62), or 30‐day mortality (6.8% vs. 5.2%, *P* = 1.00).

**Conclusion:**

The use of PCDS was associated with a lower risk of tumor ingrowth but did not improve on reintervention rates or stent patency. Both kinds of stents could be used in this group of patients.

## INTRODUCTION

Endoscopic stenting of unresectable malignant gastric outlet obstruction (GOO) is conventionally performed with uncovered duodenal stents (UCDS). Studies have shown that the use of UCDS, when compared to laparoscopic gastrojejunostomy, is associated with high rates of technical and clinical success, earlier resumption of oral diet, and shorter hospital stay.^1^ Nevertheless, these stents are prone to tumor ingrowth, leading to risk of restenosis.^2^, ^3^, ^4^ Therefore, the use of fully covered duodenal stents (FCDS) has been described. These stents may in theory reduce the chance of tumor ingrowth, but their use may be associated with increased risk of migration.^5^

Recently, partially covered duodenal stents (PCDS) have become available. The uncovered portion of the stent may help fix the stent to the gastrointestinal tract, minimizing the chance of migration, while the covered portion may reduce the chance of tumor ingrowth. Initial results of a small randomized study showed that recurrent obstructive symptoms were fewer in the PCDS group (29% vs. 3.6%, *P* = 0.0125), while no significant difference was observed in stent patency.^6^ Hence, the aim of the current study was to compare the efficacy of the PCDS vs. UCDS in an adequately powered study in patients suffering from unresectable primary malignant GOO. We hypothesize that the use of PCDS could reduce the reintervention rates when compared to UCDS.

## METHODS

This was a prospective international multicentered randomized controlled study conducted in 10 high‐volume institutions. Consecutive patients suffering from malignant GOO due to unresectable primary gastro‐duodenal or pancreatico‐biliary malignancies were recruited. Patients with obstruction of the antrum and to the 1st to 3rd parts of the duodenum were recruited (Table 1). The patients were randomized to receive endoscopic stenting with PCDS or UCDS. The local Ethics Committee approved the study protocol and the study was conducted according to the Declaration of Helsinki and the International Conference on Harmonization, good clinical practice guidelines (ICH‐GCP). The inclusion and exclusion criteria are listed in Table 2.

**Table 1:** Comparison of background demographics between the two groups

	PCDS	UCDS	*P*‐value
	*N* = 59	*N* = 56	
Age (years)	69.63 (11.64)	66.30 (15.70)	0.20
Sex (M/F)	33/26	34/22	0.71
Etiology			0.60
Gastric cancer	23	23	–
Pancreatic cancer	26	20	–
Duodenal cancer	5	3	–
Gallbladder cancer	3	4	–
Cholangiocarcinoma	0	2	–
Periampullary cancer	2	3	–
Site of stricture			0.35
Antrum	25	27	–
D1	13	13	–
D2	18	10	–
D3	3	6	–
Length of stricture (cm) median (IQR)	3 (2–5)	3.5 (3–6.9)	0.06
Types of stent used			0.41
6 cm	3	7	–
8 cm	22	15	–
10 cm	15	14	–
12 cm	19	20	–
Receiving chemotherapy	13	13	1.00

Values shown in mean (standard deviation) unless specified otherwise. IQR, interquartile range; PCDS, partially covered duodenal stents; UCDS, uncovered duodenal stents.

**Table 2:** Partially covered duodenal stent placements in malignant gastric outlet obstruction (GOO)

Inclusion criteria	Exclusion criteria
Consecutive patients ≥18 years old Confirmed unresectable intrinsic gastro‐duodenal or pancreatico‐biliary malignancies Suffering from GOO with a GOOS of ≤1^7^ Performance status ECOG ≤3	Prior metallic stent placement Severe comorbidities precluding the endoscopic procedure (such as cardiopulmonary disease, sepsis, or a bleeding disorder) Life expectancy of less than 1 month History of gastric surgery Linitus plastica Coagulation disorders Pregnancy Unable to give informed consent

ECOG, Eastern Cooperative Oncology Group; GOOS, GOO score.

### Study intervention: pyloro‐duodenal stent placement

The procedures were performed by experienced endoscopists who had inserted more than 50 duodenal stents (DS) previously. The procedures were performed under conscious sedation or monitored anesthesia. The procedures were performed with a therapeutic endoscope through the scope method. The endoscope was used to reach the site of the obstruction. The stricture was then cannulated with a 0.025″ or 0.035″ guidewire and the guidewire passed beyond the site of the obstruction. A cannula was then inserted on the guidewire for contrast injection to delineate the site and length of the stricture. A DS of adequate length to cover the entire stricture was then inserted and deployed under X‐ray and endoscopic guidance. In the case of the PCDS, the uncovered portion of the stent was placed proximal to the tumor to ensure coverage of the tumor by the covered portion of the stent. After complete deployment, contrast was injected through the stent to confirm stent patency.

### Pyloro‐duodenal stents

#### Partially covered pyloro‐duodenal stent

**Figure 1:**
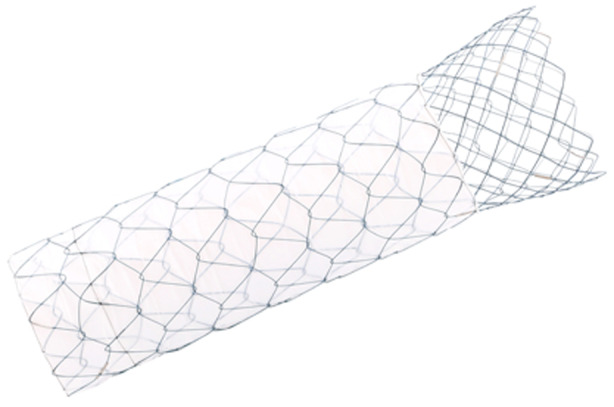
Appearance of the partially covered duodenal stent (Niti‐S COMVI – Flare; Taewoong, Gyeonggi‐do, South Korea).

**Figure 2:**
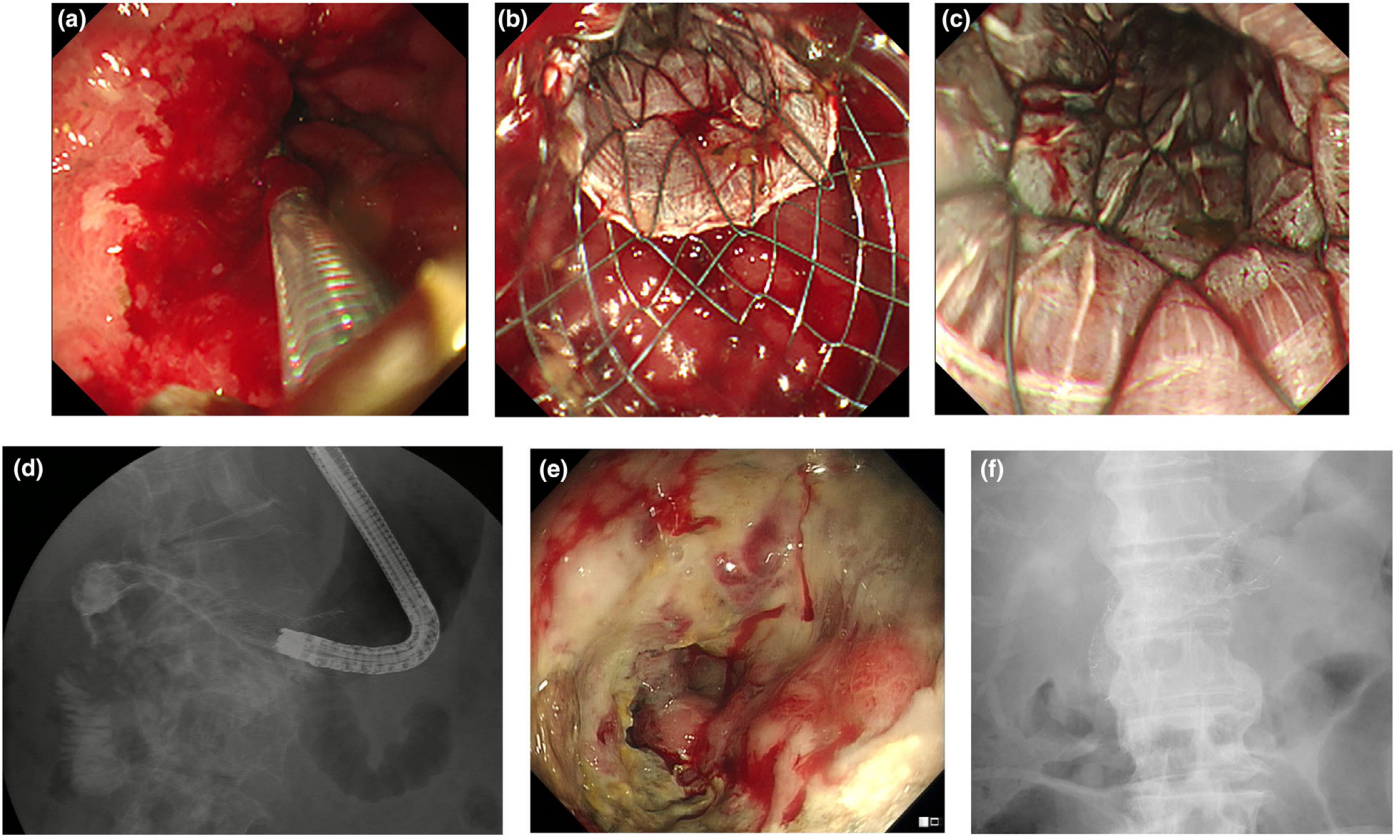
Endoscopic appearance of the partially covered duodenal stent (PCDS) after deployment. (a) Endoscopic appearance of cancer prior to stent deployment. (b) Endoscopic appearance after deployment of the stent. (c) Endoscopic appearance of the anal side of the stent after deployment. (d) Radiological image of the stent after deployment. (e) Endoscopic appearance of tumor overgrowth in the PCDS. (f) Fluoroscopic image of a patient with tumor overgrowth and insertions of an additional duodenal stent over the previously placed PCDS.

The PCDS (Niti‐S COMVI – Flare; Taewoong, Gyeonggi‐do, South Korea) used in the current study is a partially covered metallic pyloro‐duodenal stent. It consists of two portions (Figs 1, 2). The stent was 2 cm in diameter and the proximal 2 cm of the stent was uncovered and flared. This is designed to provide anchorage of the stent to surrounding tissue and reduce the risk of migration. The remainder of the stent was covered by a polytetrafluoroethylene membrane between two nitinol meshes to prevent the risk of tumor ingrowth into the stent. The stent comes in lengths of 6, 8, 10, and 12 cm.

### Uncovered pyloro‐duodenal stents

The UCDS (Niti‐S pyloric‐duodenal D stent; Taewoong) used in the current study is a standard uncovered stent made of nitinol wire, with a diameter of 2 cm and lengths of 6, 8, 10, and 12 cm. This stent is an unfixed‐cell braided stent with low axial force, high flexibility, and good conformability.

### Randomization

The patients were randomized to receive PCDS or UCDS after cannulation of the obstruction site with the guidewire. The patients and assessors were blinded to the type of stent that was inserted. The randomization codes were stratified according to the participating institution. The codes were generated by a computer in blocks of 10 and obtained via registration on the internet.

### Postprocedural management

After the procedure, the patients were fasted for 3 h and then allowed to start a fluid diet. Food intake was gradually stepped up if they tolerated the diet and did not suffer from vomiting. An abdominal X‐ray was performed the next day to confirm adequate expansion of the stent. The patients were discharged if they tolerated soft diet without vomiting.

### Outcome measurements

The primary outcome measurement is the reintervention rate. This is defined as the percentage of patients requiring additional endoscopic intervention due to stent dysfunction. Secondary outcomes included technical and clinical success, 30‐day adverse events (AE) rate, and 30‐day mortality, causes of stent dysfunction, and the duration of stent patency. Technical success was defined as successful placement of the DS across the site of obstruction, as confirmed by endoscopy or fluoroscopy. Clinical success was defined as improvement of at least 1 point in the GOO score (GOOS) within 3 days after stent insertion.^7^ Stent dysfunction is defined by restenosis of the stent due to tumor ingrowth or overgrowth, stent migration or fracture, or other causes of mechanical obstruction. Stent restenosis was considered if the patient had recurrence of obstructive symptoms, and the endoscope could not pass through the lumen of the stent. Stent migration was considered if the stent had moved from its initial position and did not cover the entire stenosis. The duration of stent patency was calculated from the time of stent placement to the time of stent dysfunction. AEs are graded according to the lexicon of endoscopic AEs.^8^

### Follow‐up

After discharge, the patients were followed up monthly in the clinic to assess for symptoms of obstruction and oral intake. Endoscopic examinations or radiological investigations were offered to patients with recurrent obstructive symptoms and a GOOS of ≤1. The patients were followed‐up to 1 year or until death.

### Sample size calculation

The sample size was calculated based on the findings of our retrospective cohort study (2016, unpubl. data). Between July 2013 and February 2016, 55 patients received endoscopic placement of a pyloro‐duodenal stent for malignant GOO. The reintervention rate of the UCDS group was 32.6% and in the PCDS group was 0%. However, in the literature the reintervention rate of PCDS was 14 to 25%. Thus, taking a mean of these values, we assume that the reintervention rate of PCDS was 10%. Assuming a 22% difference in reintervention rates between PCDS and UCDS, a two‐sided *P*‐value of 0.05, and a power of 80%, 53 patients were required in each group. Taking into account a 10% dropout rate, 58 patients were required in each group.

### Statistical analyses

All outcomes were analyzed according to the intention‐to‐treat principle. Statistical analyses were performed using SPSS 20.0 statistical software (SPSS, Armonk, NY, USA). Categorical variables were compared using Fisher's exact test or the χ^2^‐test. Continuous variables were compared between groups using the Student's *t*‐test or Mann–Whitney *U*‐test. Cumulative stent patency and patient survival times were analyzed using the Kaplan–Meier method and compared using the log‐rank test. In the analysis of the stent patency, restenosis by stent migration, tumor ingrowth/overgrowth, and stent fracture/collapse, or patient death were defined as events. Two‐sided *P*‐values of <0.05 were considered statistically significant.

## RESULTS

**Figure 3:**
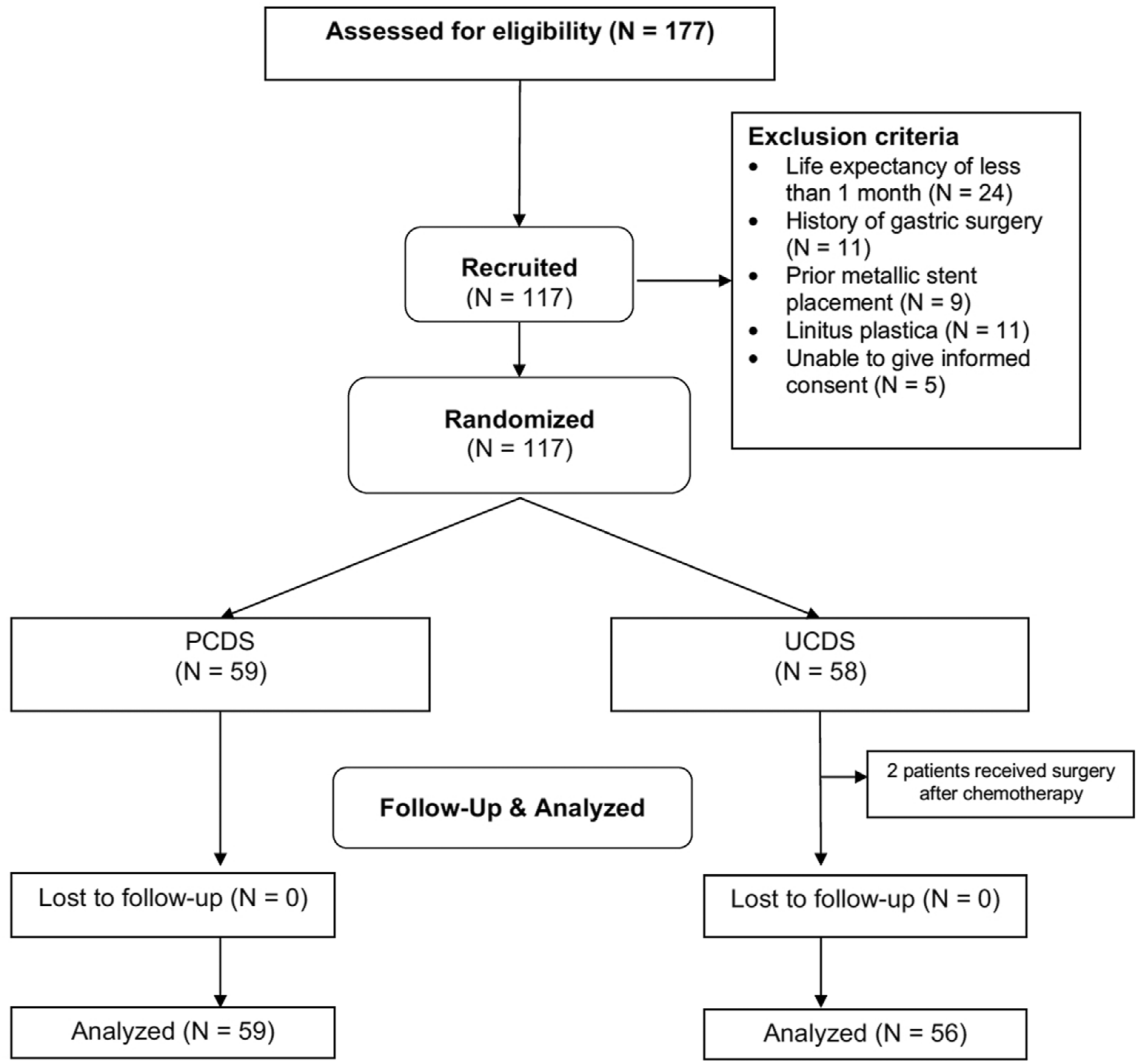
Consort flowchart. PCDS, partially covered duodenal stent; UCDS, uncovered duodenal stent.

Between March 2017 and October 2020, 177 patients were screened and 60 patients were excluded. The advent of the COVID‐19 pandemic significantly slowed down recruitment during the latter part of the recruitment period. Two patients in the UCDS group were excluded, as they received surgery after chemotherapy. Finally, 115 patients (59 PCDS, 56 UCDS) were recruited (Fig. 3). On comparison, the background demographics were not statistically different between the groups (Table 1). The majority of recruited patients suffered from pancreatic or gastric cancers and most had obstructions in the antrum and 1st part of duodenum. Two patients in the PCDS arm had strictures longer than 12 cm and required placement of two duodenal stents. There were no significant differences in the lengths of stents used in the two groups.

**Figure 4:**
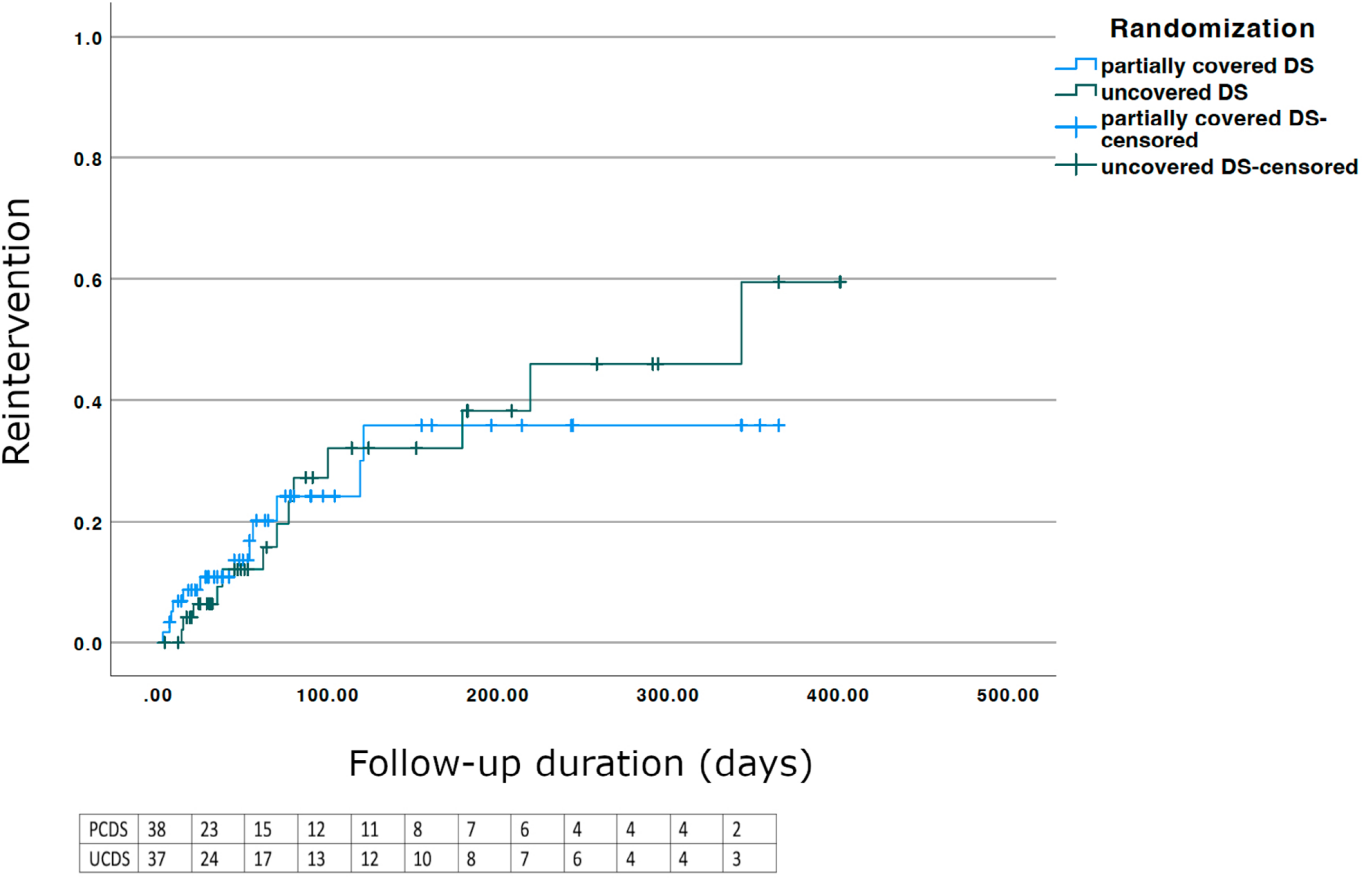
Kaplan–Meier curve showing the 1‐year reinterventions of the two groups. There were no significant differences (*P* = 0.84). DS, duodenal stent; PCDS, partially covered duodenal stent; UCDS, uncovered duodenal stent.

The 1‐year reintervention rates were not significantly different between the two groups (PCDS vs. UDCS = 12/59, 20.3% vs. 14/56, 25%, *P* = 0.84) (Table 3; Fig. 4). There was a trend to fewer patients with tumor ingrowth in the PCDS group (6/59 [10.2%] vs. 13/56 [23.2%], *P* = 0.07), but this did not reach statistical significance. All tumor ingrowth in the PCDS group occurred at the uncovered portion of the stent. Other causes of reintervention in the PCDS group included three patients with tumor overgrowth requiring an additional stent to be placed in another session, gastroparesis in two, and inadequate expansion in one patient. In the UCDS group, apart from tumor ingrowth, another patient required reintervention due to gastroparesis. On subgroup analysis of patients with gastric cancers and pancreatic cancers, no difference in reintervention rates were observed between the two groups.

**Table 3:** Comparison of outcomes between the two groups

	PCDS	UCDS	*P*‐value
	*N* = 59	*N* = 56	
Technical success (%)	59 (100.0)	56 (100.0)	1.00
Clinical success (%)	54 (91.5)	55 (98.2)	0.21
Procedural time (min)	21.5 (IQR 17–30)	20.0 (IQR 15–34.75)	0.62
Hospital stay (days)	4 (IQR 3–12)	5 (IQR 3–8)	0.81
30‐day adverse events (%)	11 (18.6)	8 (14.3)	0.62
30‐day mortality (%)	4 (6.8)	3 (5.2)	1.00
1‐year stent reintervention rate (%)	12 (20.3)	14 (25.0)	0.84
Tumor ingrowth	0	13	0.07
Gastroparesis	2	1	–
Inadequate expansion	1	0	–
Tumor overgrowth	9	0	–
Follow‐up duration (days)	58 (IQR 29–155)	76 (IQR 31–220.5)	0.27

IQR, interquartile range; PCDS, partially covered duodenal stent; UCDS, uncovered duodenal stent.

There were no significant differences in the technical success (100% vs. 100%, *P* = 1) or the clinical success rates (54/59 [91.5%] vs. 55/56 [98.2%], *P* = 0.21). In the PCDS group, three patients had clinical failure due to tumor overgrowth, two had gastroparesis, and one had inadequate expansion, as indicated above. In the UCDS group, one patient had tumor ingrowth and received an endoscopic ultrasound (EUS)‐guided gastrojejunostomy. There were also no significant differences in the duration of procedure (21.5 [IQR 17–30] vs. 20.0 [IQR 15–34.75], *P* = 0.62) and hospital stay (4 [IQR 3–12] vs. 5 [IQR 3–8] days, *P* = 0.81).

**Figure 5:**
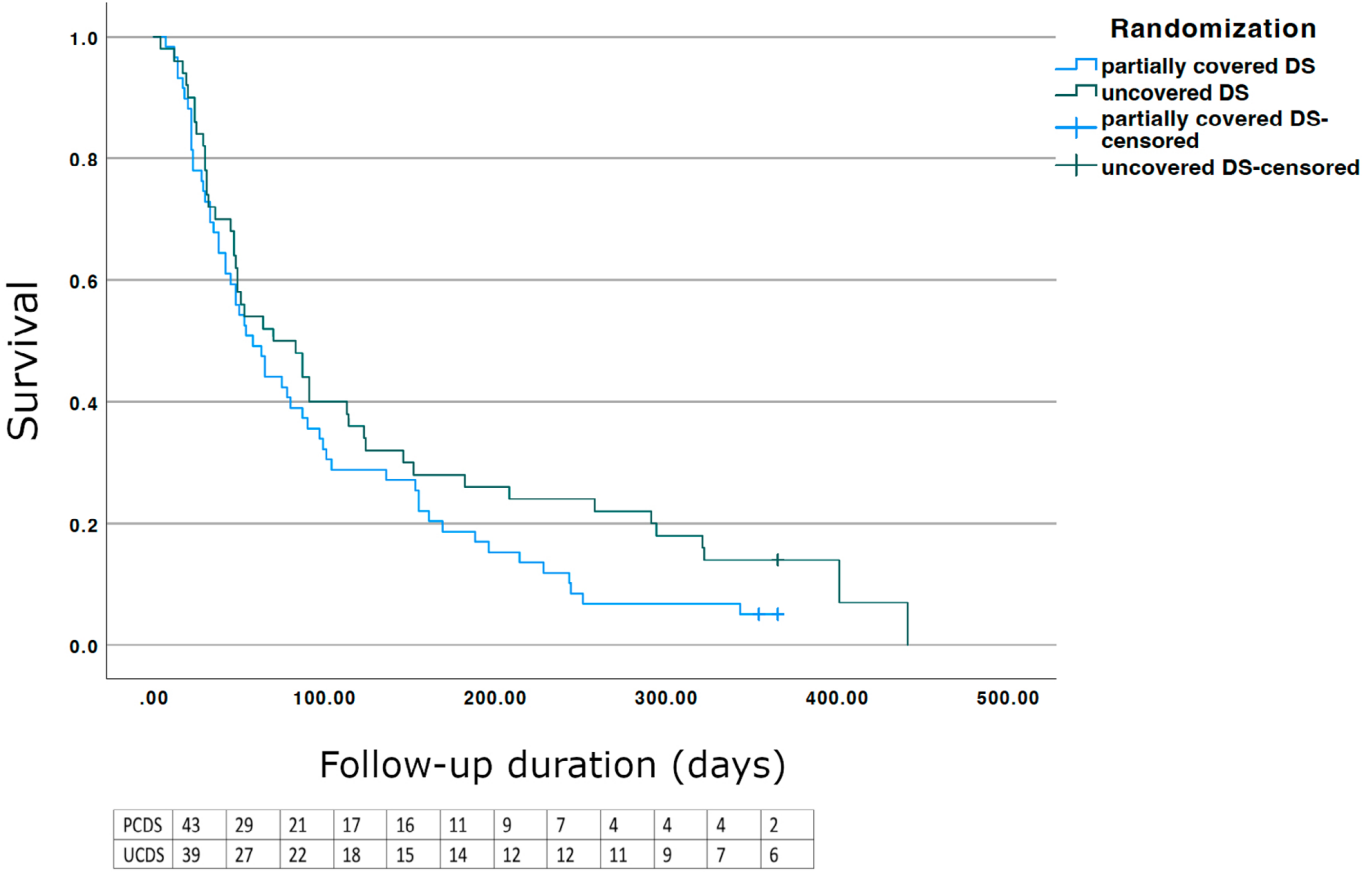
Kaplan–Meier curve showing 1‐year survival of the two groups. There were no significant differences (*P* = 0.14). DS, duodenal stent; PCDS, partially covered duodenal stent; UCDS, uncovered duodenal stent.

There were also no significant differences in the 30‐day AEs (11/59 [18.6%] vs. 8/56 [14.3%], *P* = 0.62) (Table 4) or 30‐day mortality (4/59 [6.8%] vs. 3/56 [5.2%], *P* = 1). Apart from the AEs mentioned above, in the PCDS group four patients had pneumonia and two succumbed, one patient had jaundice requiring endoscopic retrograde cholangiopancreatography (ERCP), and two had progressive deterioration of the general condition and subsequently succumbed. In the UCDS group, four patients had jaundice and two had percutaneous drainage, one had ERCP, and one refused intervention. Subsequently, two out of four of these jaundiced patients succumbed. Another patient suffered from a dense stroke with mortality, one patient had pneumonia, and another had metabolic encephalopathy. There were no significant differences in overall survival between the two groups (*P* = 0.14) (Fig. 5).

**Table 4:** 30‐day adverse events experienced in both groups

Intervention	30‐day adverse event	Severity			
		Mild	Moderate	Severe	Fatal
PCDS (*N* = 11)	Tumor overgrowth	0	2	0	0
	Gastroparesis	0	1	0	0
	Inadequate stent expansion	0	1	0	0
	Obstructive jaundice	0	1	0	0
	Pneumonia	2	0	0	2
	Generalized deterioration	0	0	0	2
UCDS (*N* = 8)	Tumor ingrowth	0	1	0	0
	Obstructive jaundice	0	2	0	2
	Stroke	0	0	0	1
	Pneumonia	1	0	0	0
	Metabolic encephalopathy	0	1	0	0

PCDS, partially covered duodenal stent; UCDS, uncovered duodenal stent.

## DISCUSSION

In the current study we were not able to demonstrate significant differences in 1‐year reintervention rates between the two groups. There was a trend to fewer patients with tumor ingrowth in the PCDS group; however, tumor ingrowth still occurred in the uncovered portion of the stent. There were also no significant differences in technical or clinical success, 30‐day AEs, or mortality.

The findings of the current study further add to the knowledge on the role of covered duodenal stents in managing malignant GOO. In 2010, Kim *et al*.^5^ compared outcomes of FCDS vs. UCDS in a randomized controlled trial and found no differences in technical or clinical successes. Stent migration occurred significantly more frequently in the FCDS group (25.8% vs. 2.85%, *P* = 0.009), while restenosis was significantly higher in the UCDS group (25% vs. 0%, *P* = 0.003). Similar findings were noted in another study by Lim *et al*.^9^ Maetani *et al*.^6^ then compared the outcomes of PCDS with UCDS. They found no significant differences in the technical or clinical success rates, but there were significantly fewer recurrent obstructive symptoms in the PCDS group (29% vs. 3.6%, *P* = 0.0125) and no difference in stent migration rates. Nevertheless, they were not able to demonstrate significant differences in stent patency due to a small sample size (*P* = 0.5175). In another study, a WAVE‐covered DS (WCS) with an antimigration design was compared with UCDS in gastric cancers with GOO.^10^ At 16‐week follow‐up, the WCS group had a significantly higher stent patency rate (68.6% vs. 41.2%, *P <* 0.01), lower stent restenosis rate (7.1% vs. 37.8%, *P <* 0.01), and longer stent patency. There were no significant differences in migration rates between the groups (*P* = 0.491). Most recently, a large‐bore PCDS stent with a diameter of 24 mm was introduced.^11^ In a retrospective study, it was shown that the patency of the large‐bore PCDS was significantly longer for extrinsic cancers (380 days vs. 121 days, *P* = 0.01), but not in intrinsic cancers (151 days vs. not reached, *P* = 0.47) when compared to 20 mm PCDS.

In the largest randomized study that included 366 patients, a PCDS with similar design to the one used in this study was compared to UCDS in patients with GOO due to both intrinsic (around 35%) and extrinsic tumors (around 64%).^12^ Interestingly, the PCDS group had significantly higher stent dysfunctions (35.2% vs. 23.4%, *P* = 0.01), stent overgrowth (*P* = 0.04), and stent migrations (*P* < 0.01). The main cause of stent dysfunction was due to stent migrations in extrinsic tumors (*P <* 0.01). While in the UCDS group, stent ingrowth was more frequent (*P <* 0.01) and time to stent dysfunction was significantly shorter (*P <* 0.01). On subgroup analysis, stent dysfunctions were significantly higher in the PCDS group for extrinsic tumors (*P <* 0.01), but not significantly different between the groups for intrinsic tumors (*P* = 0.14). The study did not report on the reintervention rates.

In summarizing the results of the above studies, whether FCDS or PCDS is beneficial for improving stent patency and reducing reintervention for malignant GOO remains controversial. The current study has a number of advantages when compared to the above studies. First, the current study did not recruit patients suffering from extrinsic tumor compression, as we were concerned with a higher risk of stent migration. This was demonstrated in Yamao *et al*.'s study.^12^ Second, we did not observe stent migration in any patient in the PCDS arm. This indicated that the uncovered portion of the stent was probably useful in anchoring the stent. However, this portion also allowed tumor ingrowth into the stent, affecting the stent patency.

Regarding limitations, although tumor ingrowth was less in the PCDS group, there were other causes of reintervention. Indicating that apart from the type of stent, other factors also contributed to the need for reintervention and an alternative method of endoscopic treatment may be needed in these patients if outcomes are to be improved. The emergence of EUS‐guided gastrojejunostomies may provide a promising alternative to duodenal stents on improving outcomes in this group of patients, as the stent is placed away from the tumor, reducing the need of reinterventions.^13^, ^14^, ^15^ Furthermore, although endoscopic stenting could alleviate symptoms of obstruction, other parameters indicating improvements in nutrition such as body mass index and laboratory data including serum albumin were not measured in this study.^16^ Finally, recent advances in chemotherapy may improve tumor response and this may affect the results of the DS.

In conclusion, the use of PCDS was associated with a lower risk of tumor ingrowth, but did not improve reintervention rates or stent patency. The technical and clinical success rates, AEs, and mortality were similar. Both kinds of stents could be used in this group of patients.

## CONFLICT OF INTEREST

Author A.Y.B.T. is an Associate Editor of *Digestive Endoscopy* and a consultant for Boston Scientific, Cook, Taewoong, Microtech, and MI Tech Medical Corporations. R.K. is a consultant for Boston Scientific, Olympus, BCM, Omega Medical Imaging, Apollo Endosurgery, Ambu, M.I.Tech, and Tigen Pharma. The other authors declare no conflict of interest for this article.

## FUNDING INFORMATION

This study was fully supported by a grant from the Health and Medical Research Fund, Hong Kong Special Administrative Region, China (Project No. 04152766).
